# Task-dependent neuromuscular adaptations in low back pain: a controlled experimental study

**DOI:** 10.3389/fnhum.2024.1459711

**Published:** 2024-09-12

**Authors:** Julien Ducas, Emile Marineau, Jacques Abboud

**Affiliations:** ^1^Department of Human Kinetics, Université du Québec à Trois-Rivières, Trois-Rivières, QC, Canada; ^2^Groupe de recherche sur les affections neuromusculosquelettiques (GRAN), Université du Québec à Trois-Rivières, Trois-Rivières, QC, Canada; ^3^Department of Anatomy, Université du Québec à Trois-Rivières, Trois-Rivières, QC, Canada

**Keywords:** high-density EMG, lumbar, pain, variability, task dependency, experimental pain

## Abstract

**Introduction:**

This study investigated the variability in lumbar neuromuscular adaptations to pain, the task dependency of pain adaptations and the effect of these adaptations on motor performance.

**Methods:**

Twenty-four healthy participants performed isometric back extension contractions at 45° and 90° trunk flexion under pain-free and experimental low back pain conditions induced by electrical stimulation. High-density surface electromyography recorded lumbar muscle activation strategies, and force steadiness was measured using a load cell.

**Results:**

While considerable variability in neuromuscular adaptations to lumbar pain was observed among participants, consistent patterns were found between tasks. In the 90° trunk flexion position, both sides exhibited greater magnitudes of pain adaptations for muscle activity redistribution in the mediolateral axis (*p* < 0.05, 86% increase) and muscle activity amplitude (*p* < 0.001, 183% increase) compared to the 45° trunk flexion position. A significant negative correlation was found between the magnitude of the mediolateral spatial redistribution of muscle activity and force steadiness on the left side (*p* = 0.045).

**Discussion:**

These findings highlight the intricate and task-dependent nature of neuromuscular adaptations to pain within lumbar muscles, and points toward a potential trade-off between pain adaptations and performance.

## Introduction

The impact of pain on human movement patterns is widely recognized, yet the intricacies of these adaptations remain elusive (Hodges and Smeets, 2015). A critical yet underexplored aspect of these adaptations is their task-dependent nature. Task dependency refers to the neuromuscular system’s response depending on the specific task being performed. Different tasks have the potential to reveal distinct pain adaptation patterns, thereby providing deeper insights into the mechanisms underlying the modulation and development of pain adaptations. For instance, studies have shown that task-specific neuromuscular adaptations to pain can vary significantly based on the degrees of freedom involved in the task (Hug et al., 2014). Tasks with limited degrees of freedom, such as knee extensions, exhibit minimal changes in muscle activity in response to pain, while tasks with higher degrees of freedom, such as bilateral leg squats, can lead to significant reductions in muscle activity in affected muscles (Hug et al., 2014). In complex neuromuscular systems, such as the trunk muscles, the task dependency of pain adaptations remains inconclusive. A recent meta-analysis hypothesized that the high heterogeneity of study results could be attributed to the task-dependent nature of neuromuscular adaptations to experimentally induced lumbar pain (Devecchi et al., 2022). Yet, studies comparing neuromuscular adaptations across different tasks observed no significant differences (Hodges et al., 2013; Ducas et al., 2024). This absence of differences between tasks is likely due to the substantial variability in individual responses to pain (Hodges et al., 2013, Ducas et al., 2024). This variability can mask consistent patterns or differences between tasks, making it challenging to detect statistically significant effects of task dependency. Our previous research demonstrated that, even when controlling for factors such as pain intensity, location, and duration using experimental pain, lumbar pain adaptations were highly variable among individuals, resulting in minimal overall effects (Ducas et al., 2024). Similarly, Hodges et al. (2013) found that acute lumbar muscle pain led to unique activation patterns in trunk muscles among participants resulting in no significant overall main effect for task differences (Hodges et al., 2013). This inter-individual variability in lumbar pain adaptations may be attributed to the significant redundancy in motor function within these muscles, providing multiple available adaptation strategies (Hodges and Tucker, 2011; Saito et al., 2023). To alleviate pain, participants may redistribute muscle activity both within specific muscles (e.g., motor unit territories of the longissimus muscle) and across different muscle groups (e.g., erector spinae and multifidus) (Hodges and Tucker, 2011; Ducas et al., 2024; Abboud et al., 2020).

The inconsistent findings across studies on task dependency (Hug et al., 2014; Ducas et al., 2024; Hodges et al., 2013), coupled with significant inter-individual variability in lumbar pain adaptations, indicate that our understanding of how different tasks influence pain adaptations in the lumbar region remains incomplete. Current assessment methods, which often focus on the direction of pain adaptations, such as increases or decreases in muscle activity, may not fully capture the complexity of pain adaptations. This gap highlights the need for new approaches such as evaluating the magnitude of pain adaptations. The assessment of pain adaptation magnitude could account for individual differences in adaptations and provides a holistic view of the overall adaptation patterns. Moreover, the magnitude of the change could be important, as compensatory adaptations, regardless of their direction, have been observed to persist even after pain resolution (Summers et al., 2019; Tucker et al., 2012; Gallina et al., 2018).

The initial aim of this study was to descriptively show inter-individual variability in adaptations to experimental pain. Subsequently, the primary objective was to assess how different tasks influenced the magnitude of pain adaptations. Additionally, the study aimed to explore whether a greater magnitude of pain adaptations was correlated with a decreased ability to achieve the task’s goal, measured by force steadiness. We hypothesize that pain adaptations will exhibit significant inter-individual variability, consistent with findings from previous studies (Hodges et al., 2013; Ducas et al., 2024). We hypothesized that the magnitude of adaptations to pain would depend on the task being performed. Moreover, we hypothesized that greater magnitude of pain adaptations would result in a significant decrease in force steadiness.

## Methods

### Participants

Twenty-four adult participants (12 men and 12 women) aged 25.83 ± 4.95 years participated in this study. The mean height, weight, and BMI of the participants were, respectively, 170.44 ± 7.63 cm, 69.57 ± 10.14 kg and 23.88 ± 2.66 kg/m^2^. A total of 24 participants was required to achieve a statistical power of 0.8 and an alpha of 0.05 for a two tail pairwise t-test with a moderate effect size using G*Power 3.1 (Faul et al., 2007). Exclusion was based on the following criteria: the presence of low back pain in the past year; spinal surgery; inflammatory arthritis of the axial skeleton; advanced osteoporosis; pregnancy; heart pacemaker; skin leisure; abnormal skin sensitivity and severe and incapacitating pain limiting the ability to perform the evaluation protocol in the laboratory. The project received approval from the Research Ethics Board for human research of the “Université du Québec à Trois-Rivières” (CER-22-287-07.07). All participants gave their written informed consent, acknowledging their right to withdraw from the experiment without consequences. The study was conducted according to the principles of the Declaration of Helsinki (2013).

### Experimental protocol

To answer the objectives of the study, all participants were tested in one session. Initially, sociodemographic information was collected, including age, sex, weight and height. After this assessment, isometric back extension contractions were performed in two positions (trunk flexion at 45°, and trunk flexion at 90°) during which activation of the lumbar extensor muscles (LEM) was evaluated by using high-density surface electromyography (HDsEMG). For each position, while participants contracted their back muscles, they experienced two conditions. One with experimental pain (electrical stimulus) in the lumbar region (lumbar pain), and one without a painful stimulus (baseline). Participants first underwent the baseline condition and then the painful one (Gallina et al., 2018). Randomization was performed for the order of the position. In addition, a 5-min rest period was provided between positions to minimize muscle fatigue.

### Trunk positions

**Figure 1:**
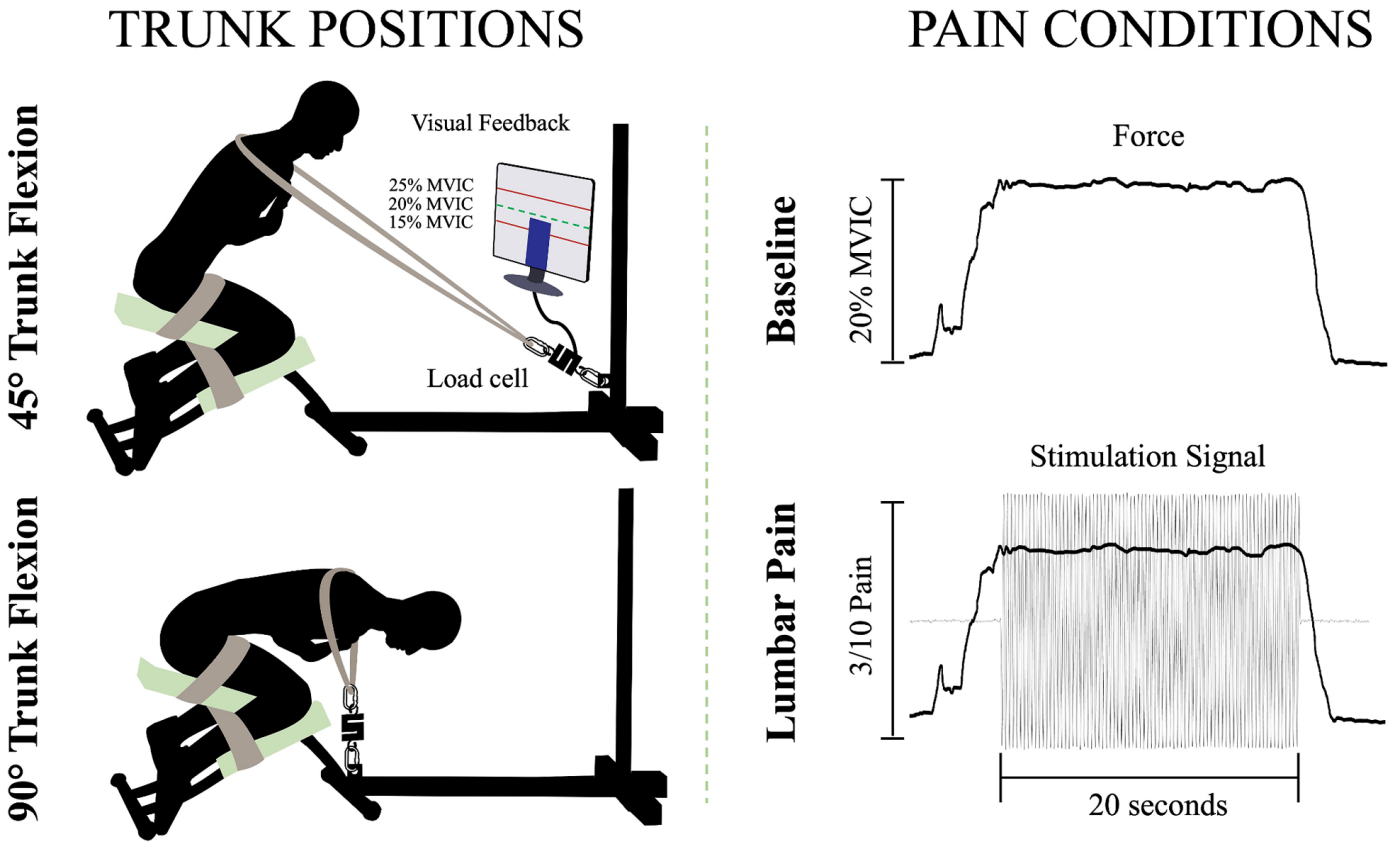
Illustration of the isometric trunk extension protocol in two different positions and the two pain conditions: 45° trunk flexion (upper left figure), 90° trunk flexion (bottom left figure), baseline condition (upper right figure) and lumbar pain condition (bottom right figure); MVIC, maximal voluntary isometric contraction.

To perform the isometric contractions, participants were installed on a custom-made apparatus (Figure 1). The participants’ thighs and calves were fastened to the apparatus with straps to prevent any forward movement of the pelvis and knee flexion. While on this apparatus, participants were instructed to adopt two distinct trunk positions while crossing their arms over their chests. For the two positions, 45° of trunk flexion and 90° of trunk flexion, participants were semi-seated with their knees flexed at ∼75°. The aim of these positions was to modify the length (Macintosh et al., 1993) and the orientation (Harriss and Brown, 2015) of the LEM fibers, thus altering the mechanical advantage of these fibers. During the trunk flexion at 45° position, it is believed that the LEM fibers are positioned at a favorable length, between the maximum stretch and the maximum shortening of the muscle. This alignment enhances force generation, in accordance with the principles outlined in the force-length relationship (Gordon et al., 1966). In addition, the muscle fibers of the LEM are oriented posteriorly and caudally (Harriss and Brown, 2015), thus inducing posterior shear forces to facilitate spine extension. Conversely, in the trunk flexion at 90° position, the LEM fibers are aligned parallel to the spine (Harriss and Brown, 2015) and are stretched (Macintosh et al., 1993). Therefore, muscle fibers lose their mechanical advantage to generate force posteriorly and potentially have a reduced ability to perform the needed contraction. A digital inclinometer (precision of ±0.1°, model 40–6,067, Johnson Level & Tool Mfg. Co., Inc. Mequon, WI) placed on the L3 spinous process was used to measure the trunk flexion angle for each position. Then, a belt was adjusted to the correct length, placed on the shoulders of the participants, and attached to the apparatus. Linked between the belt and the apparatus, a load cell (precision of ±0.05%, Model LSB350; Futek Advanced Sensor Technology Inc., Irvine, CA, United States) recorded the force produced during isometric back extension contractions.

### Isometric back extension contraction tasks

For each position, three maximal voluntary isometric contractions (MVIC) of the trunk in extension were held for 3 s. A one-minute rest was provided between each MVIC trial. Then, for the submaximal isometric back extension contractions, participants were asked to produce 20% of the highest MVIC of the trunk at each of the two different positions. This ensured that the target force exerted by the participants in each position is participants and position dependent. The force percentage was chosen to mimic the intensity of LEM contractions experienced during everyday activities (Abdoli-E et al., 2006). Participants were looking at a computer screen to visually monitor their force production during the contractions. For both the baseline condition and the painful condition, participants were asked to maintain the task goal for 20 s. For the lumbar pain condition, upon achieving and sustaining the target force, a 20-s electrical stimulation was administered (Figure 1). This setup aligns with previous study (Ducas et al., 2024).

### Experimental low back pain

**Figure 2:**
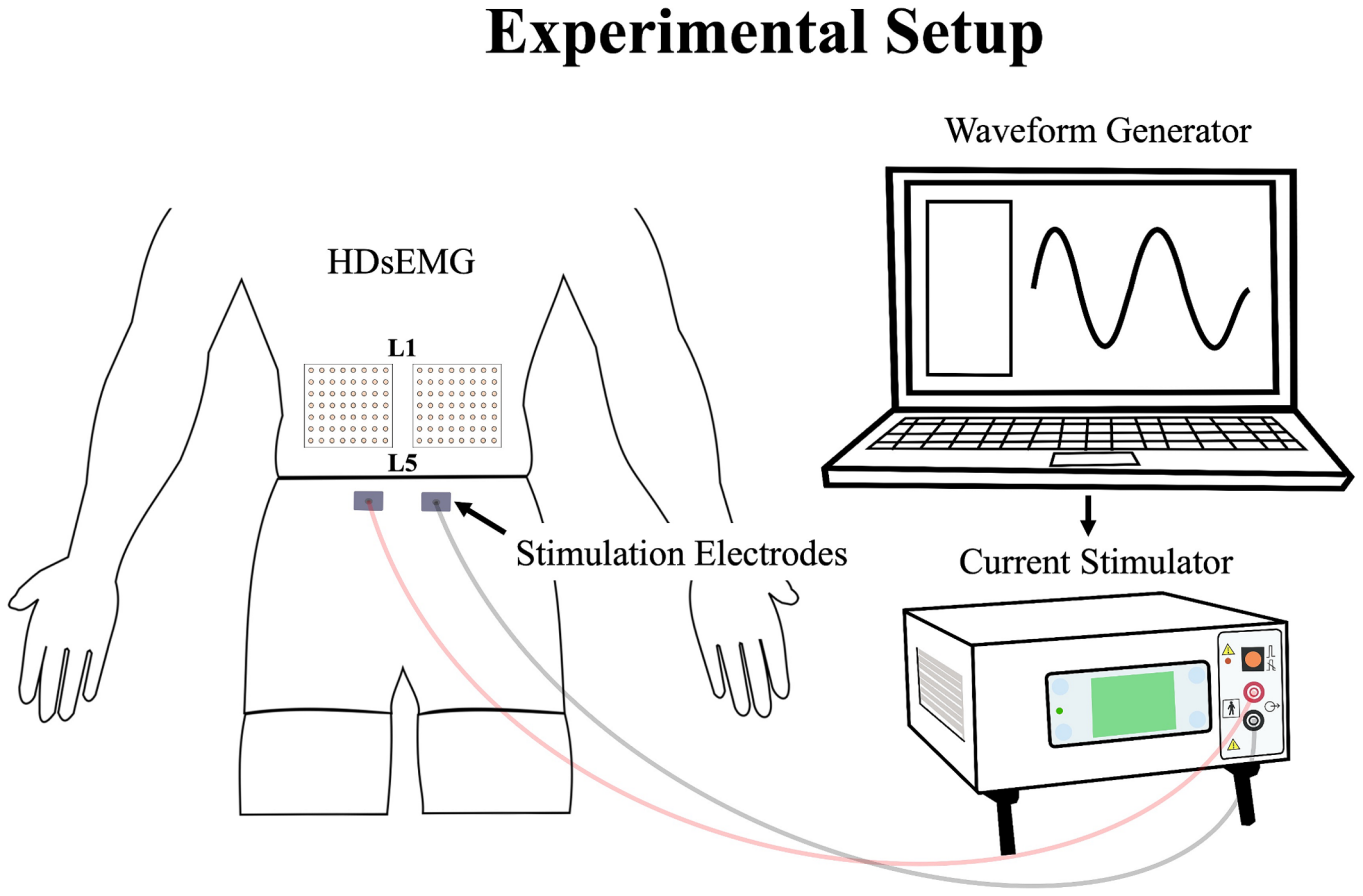
Illustration of the experimental setup, demonstrating the placement of the HDsEMG electrodes and electrical stimulation electrodes; HDsEMG, High-Density surface electromyography.

Low back pain was investigated experimentally, using electrical stimulation due to its precise control over both intensity and duration of pain. The electrical stimulation signal was generated using Spike2 software and a CED Power1401 data acquisition system (Cambridge Electronic Design Ltd., Milton, Cambridge, United Kingdom) and was delivered through a DS5 constant current stimulator (Digitimer, Letchworth Garden City, Hertfordshire, United Kingdom). The stimulation signal consisted of a sinusoidal waveform with a frequency of 4 Hz, known to selectively trigger C-fibers of the nociceptor fibers (Jonas et al., 2018) and generate negligible artifacts on electromyography signals, allowing accurate quantification of muscle activity (Gallina et al., 2021). See section *Stimulation-Induced Artifacts on HDsEMG Signals* for further details. Moreover, a 4 Hz sinusoidal waveform limits habituation of pain intensity within a timeframe of 60 s or less (Gallina et al., 2021). Electrical stimuli were administered through two surface electrodes (2*4 cm, Anself) placed on the posterior superior iliac spine, identified by palpation (Figure 2). The positioning of the electrodes is detailed in our previous study (Ducas et al., 2024). The amplitude of the waveform’s stimulation was individually adjusted to standardize pain intensity, determined through an ascending protocol. For protocol details, see our previous study (Ducas et al., 2024). The protocol was stopped when participants perceived moderate pain intensity of 3/10 measured using a verbal numerical rating scale (NRS), anchored between 0 (no pain) and 10 (worst pain imaginable). A 3/10 painful stimulus was used, as it has previously proven effective in eliciting significant neuromuscular adaptations (Gallina et al., 2018; Gallina et al., 2021; Summers et al., 2019; Ducas et al., 2024). This level of stimulation was chosen to balance effectiveness with safety, ensuring minimal risk and adhering to ethical standards. When the pain intensity target was reached, the amperage was recorded and used for the 20-s submaximal contractions. If a participant reported pain levels exceeding or falling below 3/10 after a trial, the stimulation intensity was reevaluated, and the trial was repeated. Participants had to rate their perceived pain using the NRS.

### Data collection

The myoelectric activity of the right and left LEM was recorded during all maximal and submaximal contractions with two HDsEMG grids. The grids included 64 individual electrodes arranged into 8 columns and 8 rows spaced by 10 mm (semidisposable adhesive matrix; model ELSCH064, OTBioelettronica, Torino, Italy). Differential signals were calculated from HDsEMG by subtracting consecutive monopolar signals along the craniocaudal direction. This process led to the final configuration of the grid of 7 × 8 channels. To optimize and standardize the electrical impedance under the grids and electrodes sites, the skin of the participants was shaved, cleaned with a fine grade sandpaper (Red DotTrace Prep; 3 M, St. Paul, MN) and with alcohol (ethyl alcohol 70%). Then the HDsEMG grids were positioned so that the columns were oriented to align with the direction of the LEM fibers. To minimize variation, a single researcher conducted the grid placement for all participants. The central point of each grid was located at the spinous process of L3, and the inner edge of the grids was positioned approximately 1 cm from the spinous process of L3. The grid spanned approximately from the L1 to the L4-L5 region (Figure 2). It covered the two primary LEMs: the superficial multifidus and the erector spinae muscles, arranged from medial to lateral relative to the centerline of the spine, respectively (Gallina et al., 2024). The reference electrode was placed on the right iliac crest of each participant. To avoid signal saturation during electrical stimulation, the bipolar HDsEMG signals were amplified by a factor of 100. They were then digitized at a sampling rate of 2048 Hz using a 12-bit A/D converter (128-channel EMG-USB; OTBioelettronica) with a bandwidth of 10–500 Hz (−3 dB). Simultaneously with the recording of HDsEMG signals, a trigger signal was also captured to precisely identify the start and end of the electrical stimulation. This trigger signal ensured accurate synchronization between the stimulation events and the corresponding HDsEMG recordings. Additionally, the force signal generated during the isometric contractions was captured using a load cell and collected through the HDsEMG amplifier system.

### Data analysis

The signals were imported and analyzed using Matlab (v.2023b; TheMathWorks, Natick, MA). Throughout all isometric trunk extension contractions, HDsEMG data were gathered and analyzed independently for both the left and right sides. Then, the HDsEMG signals were filtered using an 8th-order Butterworth filter with a frequency range of 30–400 Hz. This filter has proven to minimize stimulation-induce artifact of 4 Hz electrical stimulation on HDsEMG channels (Gallina et al., 2021). In addition, a 2nd-order Butterworth notch filter was used to eliminate interference from the 60 Hz power lines and its harmonics. For the recording during all isometric trunk extension contractions, a comprehensive visual examination of the raw HDsEMG signals was conducted, which led to the identification of electrodes exhibiting contact issues or artifacts induced by stimulation. To further evaluate the presence of stimulation-related artifacts, amplitude spectra were computed for all electrodes and for each condition using a Fast Fourier transform and were visually screened. For electrodes that had such problems, a reconstruction technique was applied by interpolating data from neighboring electrodes. This approach effectively addressed the problematic electrodes and ensured the integrity of the HDsEMG signal data. Interpolation was performed only for electrodes with vertically adjacent neighbors. Consequently, electrodes in the first or last rows, which lacked neighboring electrodes, were removed rather than interpolated. If these electrode artifacts were observed on more than 10% of all electrodes, the recording was excluded from the analysis (Gallina et al., 2022). The 10 middle seconds of each signal recording were analyzed and segmented into consecutive windows of 0.5 s. Force signals underwent a low-pass filtering at 10 Hz using a 2nd-order Butterworth filter.

Dependent variables were calculated for each pain condition (baseline and lumbar pain) and for each position (45° trunk flexion and 90° trunk flexion). Specifically, pain intensity, HDsEMG variables, and force steadiness were computed. Regarding the dependent variable of HDsEMG, Muscle activity amplitude corresponded to the mean of the root mean square (RMS) in all filtered channels, which is then normalized using the mean RMS of the MVC in its corresponding position. To determine the spatial distribution of LEM activity, the means of the mediolateral and craniocaudal coordinates of the centroid were calculated as previously described (Farina et al., 2008; Falla et al., 2009; Madeleine et al., 2006; Abboud et al., 2014).

**Figure 3:**
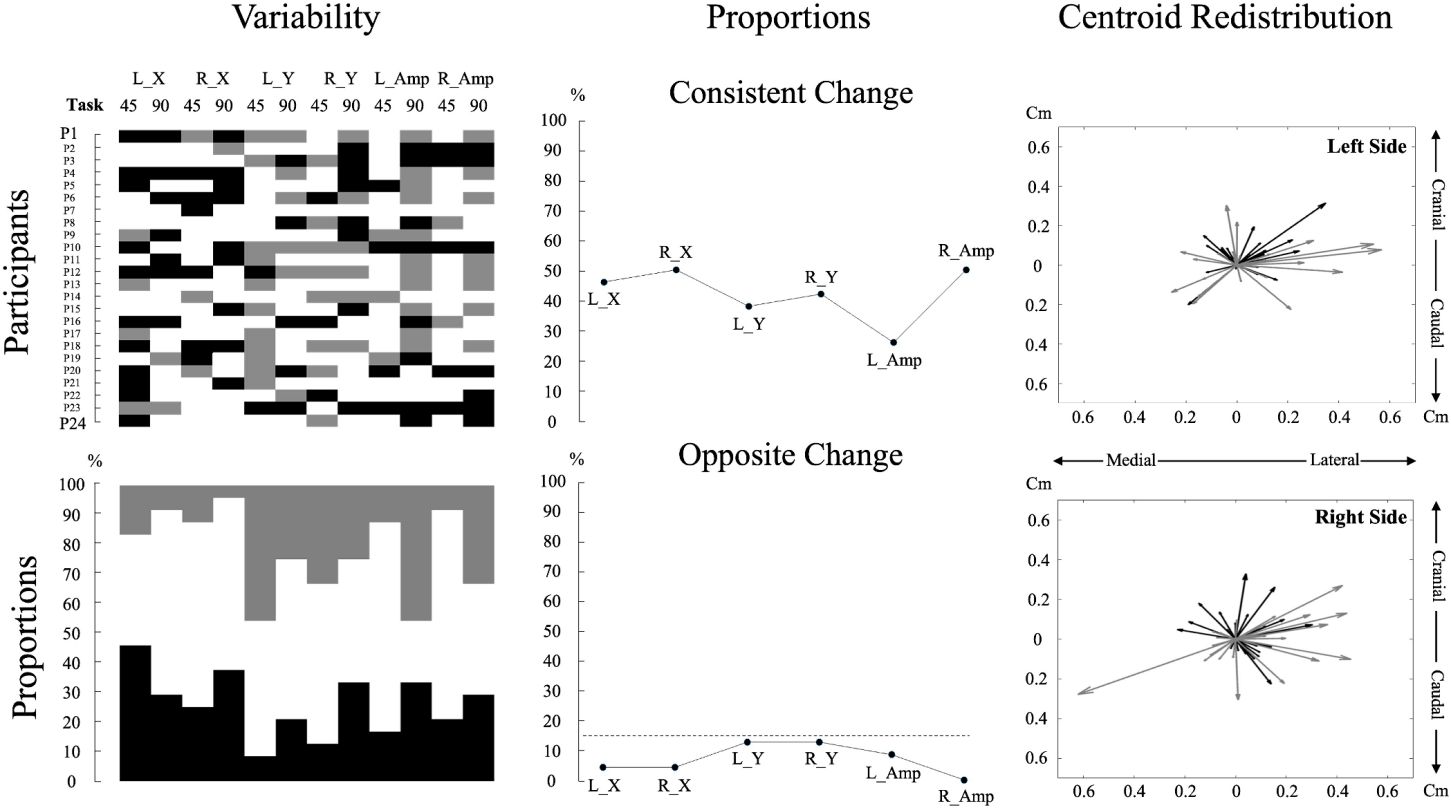
Individual data and analyses of muscle activity amplitude and redistribution changes during pain. The top left subplot displays individual participant data for changes in muscle activity amplitude and redistribution during pain across the two tasks for each variable. For muscle activity amplitude, black denotes an increase of more than 15%, gray means a decrease of more than 15%, and white indicates no change from the baseline (Hodges et al., 2013; Chapman et al., 2008). For muscle activity redistribution, black represents a shift to a more lateral or caudal position, while gray represents a shift to a more medial or cranial position relative to the baseline. A shift was considered significant if it exceeded one standard deviation from the baseline, with the standard deviation calculated based on the changes observed across all participants. This approach was used due to the lack of a predefined threshold for significant muscle redistribution. The bottom left subplot, using the same color code as for the top left subplot, illustrates the percentage of individual participants encountering an increase, decrease, or no change for each variable and task. The top middle subplot depicts the proportion of participants displaying consistent changes, characterized by similar directional changes (e.g., an increase in muscle activity amplitude in both tasks). The bottom middle subplot represents the proportion of participants displaying contradictory changes, reflecting opposite directional changes (e.g., increase in muscle activity amplitude in one task and decrease in the other) for each variable. The dashed line represents a threshold of 15%. The two right subplots portray centroid displacements from the baseline as vectors for all participants, offering a visual representation of the variability in overall directional displacement. The center of the plot (coordinates 0,0) represents the baseline condition. A black vector represents centroid displacements in the 45° trunk flexion, while a gray vector represents displacements in the 90° trunk flexion. L_X: Left side mediolateral muscle activity redistribution; R_X: Right side mediolateral muscle activity redistribution; L_Y: Left side craniocaudal muscle activity redistribution; R_Y: Right side craniocaudal muscle activity redistribution; L_Amp: Left side muscle activity amplitude changes; R_Amp: Right side muscle activity amplitude changes; 45: 45° trunk flexion; 90: 90° trunk flexion.

To describe variability in pain adaptations between individuals, data on muscle activity amplitude and redistribution were graphically displayed for each participant in both tasks (see Figure 3 for details). These analyses aimed to highlight inter-individual variability and were used as a precursor to the assessment of neuromuscular adaptation magnitude.

To evaluate the magnitude of neuromuscular adaptations to pain, the centroid coordinates collected during the pain condition were subjected to a subtraction process from the one collected during the baseline condition. The resulting differences were presented into absolute values to assess the magnitude rather than the direction of redistribution. For muscle activity amplitude, the percentage of change from the baseline condition was assessed and presented into absolute values to assess the magnitude of neuromuscular amplitude adaptations to pain.

To assess the ability to maintain the task goal of 20% of the MVIC, force steadiness was used as a performance metric and represented by the standard deviation (SD) of the force during the isometric back extension contraction. To evaluate how the magnitude of neuromuscular adaptations to pain influences performance, the force steadiness during the lumbar pain condition was subtracted from the baseline condition. However, unlike the HDsEMG data, these differences were not converted into absolute values. This approach was designed to discern whether there was an increase or decrease in the ability to produce force.

### Statistical analyses

The statistical analyses were conducted with SPSS Statistics for Mac, version 28 (SPSS Inc., IBM Corp., Armonk, NY, United States). The selection between parametric and nonparametric tests was based on the normal distribution of the data, determined by both the Kolmogorov–Smirnov test and visual inspection.

For normally distributed dependent variables, a pairwise t-test was used to compare the magnitude of neuromuscular adaptations between trunk positions. For non-normally distributed variables, the Wilcoxon test was used. Effect sizes were quantified using Cohen’s d for normally distributed variables and *r* = $\frac{\left|Z\right|}{\sqrt{n_{x}+n_{y}}}$ for non-normally distributed variables (Pallant, 2020). Both value can be interpreted using Cohen D, with values of 0.2, 0.5, and 0.8 indicating small, medium, and large effect sizes, respectively (Cohen, 2013). The findings were reported using mean values and standard deviations (SD) for normally distributed variables and median and quartiles (Q1, Q3) for non-normally distributed variables.

Furthermore, Pearson’s correlation was performed to investigate the relationship between neuromuscular adaptations magnitude and force steadiness, independent of trunk position. Consequently, data from both trunk positions were pooled together for the correlation analysis. This integration was made possible through the normalization of both SD and muscle activity amplitude and distribution per position. This approach effectively eliminated the positional effect, allowing the pooling of data. A significance level of *p* < 0.05 was considered for all statistical assessments.

### Stimulation-induced artifacts on HDsEMG signals

To validate the negligible impact of stimulation-induced artifacts on HDsEMG signals, an assessment was conducted with the initial 10 participants. HDsEMG recordings were acquired at rest, in a neutral trunk position without stimulation, and during stimulation. The same method of signal filtering for isometric contraction was used excluding only electrodes with contact issues or baseline noise. The effect of stimulation on EMG amplitude artifacts was statistically evaluated using the Wilcoxon test, comparing RMS values under two conditions: at rest without stimulation and at rest with stimulation. Median values were reported. To identify stimulation artifacts, a criterion was established that required a statistically significant difference (p < 0.05) and a median increase of at least 2 *μ*V. This threshold was chosen considering that the average baseline noise should not exceed 3–5 *μ*V (Konrad, 2005), making a 2 *μ*V change a conservative criterion (Ducas et al., 2024).

The results did not show artifacts induced by stimulation on any electrodes, as indicated by the absence of a median increase in RMS of at least 2 μV and a statistically significant difference with *p* < 0.05. The average (max:min) median increase across all channels during lumbar pain on the left side was 0.401 (1.079:−0.672) μV, and was 0.460 (1.849:−0.594) *μ*V on the right side.

## Results

Pain intensity did not differ across positions [df (23) *t* = 0.247, *p* = 0.807, Cohen, d = 0.050]; (45°: 3.10 ± 0.44; 90°: 3.07 ± 0.53). Neuromuscular adaptations to lumbar pain exhibited considerable variability between participants, with each participant displaying a unique pattern (Figure 3).

### Effect of tasks

#### Mediolateral redistribution magnitude

**Figure 4:**
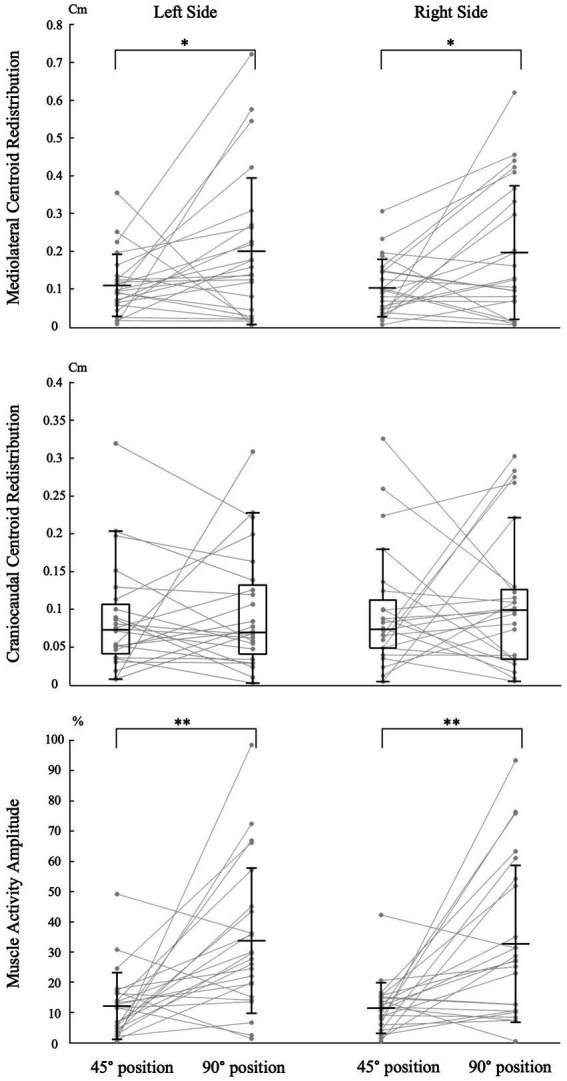
Impact of trunk position on neuromuscular adaptation magnitude. Individual data points are connected by lines for both trunk positions. The symbols “*” and “**” indicate statistical significance with *p*-values of <0.05 and < 0.001, respectively. The labels “45° position” and “90° position” correspond to the trunk flexion positions of 45° and 90°, respectively. Mean and standard deviation are presented for muscle activity amplitude and centroid redistribution in the mediolateral axis. For centroid redistribution on the craniocaudal axis, median and quartiles are presented using a box plot.

Trunk position had a significant impact on the magnitude of mediolateral muscle activity redistribution for both the left [*t* (23) = −2.110, *p* = 0.046, Cohen d = 0.431] and right [*t* (23) = −2.485, *p* = 0.021, Cohen d = 0.507] sides (Figure 4). Refer to Table 1 for mean scores (SD).

**Table 1:** Trunk position impact on neuromuscular adaptation magnitude: mean and median scores.

Variables	Sides	Positions	Mean score ± SD	Median score (Q1, Q3)
Mediolateral redistribution magnitude (cm)	Left	45°	0.105 ± 0.082	
		90°	0.195 ± 0.192	
	Right	45°	0.101 ± 0.076	
		90°	0.189 ± 0.177	
Craniocaudal redistribution magnitude (cm)	Left	45°		0.074 (0.040, 0.110)
		90°		0.071 (0.039, 0.135)
	Right	45°		0.074 (0.048, 0.119)
		90°		0.100 (0.033, 0.129)
Muscle activity amplitude magnitude (%)	Left	45°	12.125 ± 11.132	
		90°	33.972 ± 24.293	
	Right	45°	11.308 ± 8.326	
		90°	32.370 ± 25.737	

#### Craniocaudal redistribution magnitude

Trunk position had no significant impact on the magnitude of craniocaudal muscle activity redistribution for both the left (Z = −0.257, *p* = 0.797, Cohen d = 0.037) and right (Z = −0.486, *p* = 0.627, Cohen d = 0.070) sides (Figure 4). Refer to Table 1 for median scores (Q1, Q3).

#### Muscle activity amplitude magnitude

Trunk position had a significant impact on the magnitude of muscle activity amplitude for both the left [*t* (23) = −3.771, *p* < 0.001, Cohen d = 0.770] and right [*t* (23) = −3.717, *p* < 0.001, Cohen d = 0.759] sides (Figure 4). Refer to Table 1 for mean scores (SD).

### Correlation between pain adaptations magnitude and force steadiness

**Figure 5:**
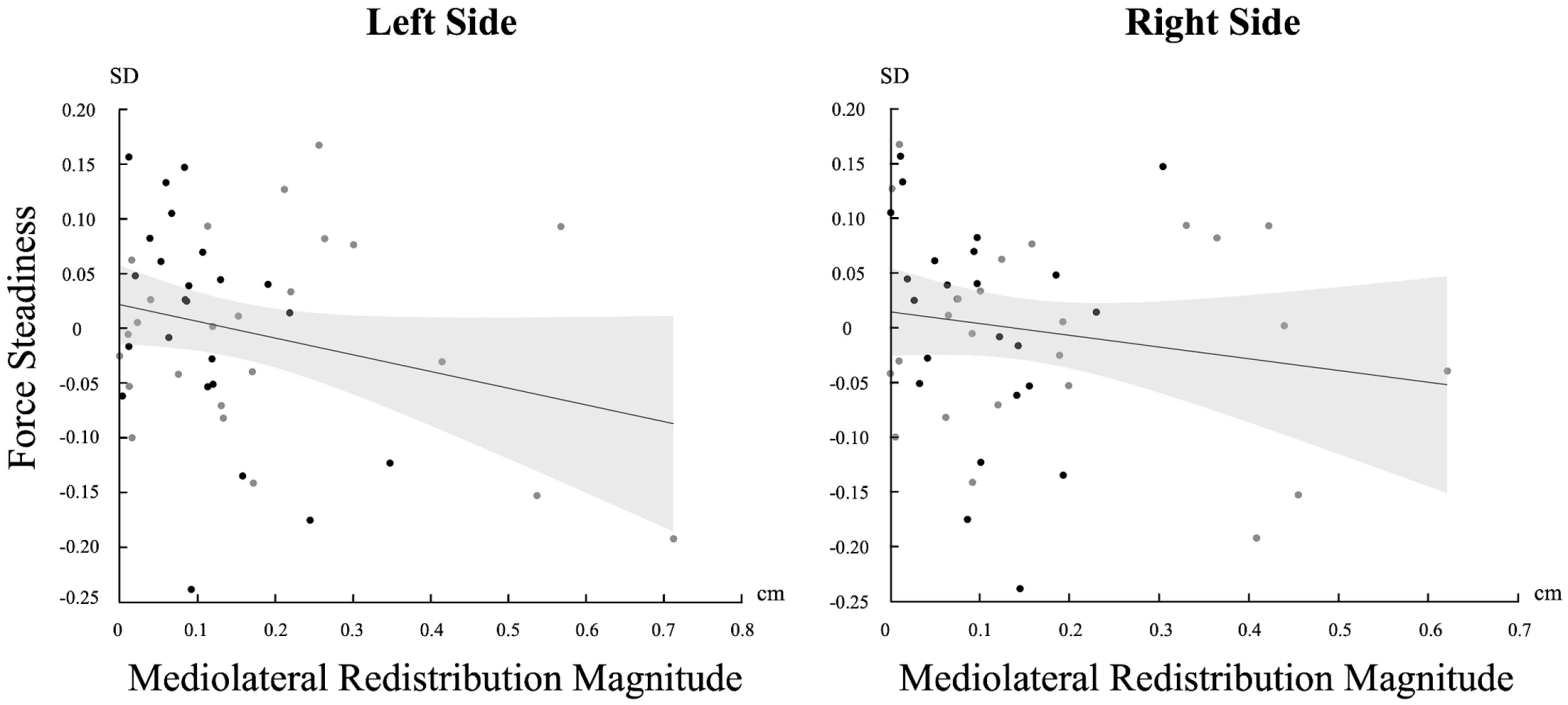
Correlation between the standard deviation (SD) of force and the magnitude of change in the mediolateral axis for both sides (Left and Right). Light gray represents confidence intervals.

A significant negative correlation was found between the magnitude of muscle activity redistribution in the mediolateral axis and force steadiness on the left side [*r* (46) = −0.247, *p* = 0.045]. Moreover, a similar trend was observed on the right side, although not statistically significant [*r* (46) = −0.161 *p* = 0.137] (Figure 5). For a complete overview of the correlations with other variables, refer to Table 2.

**Table 2:** Pearson correlation between the magnitude of pain adaptations and force steadiness.

Variables	Mediolateral		Craniocaudal		Muscle activity amplitude	
Side	Left	Right	Left	Right	Left	Right
Pearson correlation (*N* = 48)	**−0.247**	−0.161	−0.062	−0.151	0.155	0.090
*p*	**0.045**	0.137	0.338	0.153	0.146	0.272

*Significance highlighted in bold.

## Discussion

This study assessed the impact of trunk position on neuromuscular adaptations to pain and its relationship with maintaining task goal measured through force steadiness. The results initially showed significant variability between individuals in adapting to lumbar pain, with each participant using a distinct adaptation pattern. Furthermore, we observed that participants used similar muscle activation strategies in both tasks. Despite the predominance of consistent adaptation directions, the study showed that the influence of pain on both the adaptation of muscle activity amplitude and redistribution (mediolateral) magnitude differs depending on the task. Greater magnitude of pain adaptations was evident when the trunk was in mechanical disadvantage. Additionally, the results showed a correlation between the magnitude of muscle activity redistribution to pain along the mediolateral axis and the ability to maintain the task goal, where greater pain adaptations lead to a decreased in performance. This points toward a potential trade-off, between pain adaptations and performance.

### Variability in pain adaptation

As anticipated and consistent with prior research, this study found high inter-individual variability in pain adaptations even when controlling for pain intensity, location and duration. To adapt to pain, participants may have leveraged the versatility of the trunk by redistributing muscle activity to other muscles, which were not directly measured in this study, resulting in variable changes in muscle activity amplitude. Hodges et al. (2013) found similar results in the lumbar region using acute lumbar muscle pain (hypertonic saline injection) during dynamic back extension and flexion tasks while bilaterally measuring the muscle activity of the rectus abdominis, internal oblique, external oblique, latissimus dorsi and the erector spinae in both the thoracic and the lumbar region. They observed variability in adaptations within individuals resulting in a null main overall effect for pain adaptations.

As for the lack of a uniform directional muscle activity redistribution to pain, it may be explained by variations in participants’ anatomy and anthropometry. These variations, which alter individual biomechanics (Putzer et al., 2016; Onyemaechi et al., 2016), could influence the selection of the pain adaptation strategy. This pattern of non-directional adaptations of lumbar muscle redistribution has also been observed during endurance tasks (Sanderson et al., 2019). Sanderson et al. (2019) demonstrated that both healthy individuals and those with low back pain exhibited an increased magnitude of muscle activity redistribution in response to heightened lumbar muscle fatigue. However, the specific direction of this redistribution varied between individuals (Sanderson et al., 2019).

Despite high inter-individual variability, descriptive analysis of the current study revealed that less than 15% of adaptations were in opposite directions between tasks for both muscle activity amplitude and redistribution, indicating minimal intra-individual variability between tasks. These findings align with those of Hodges et al. (2013), who reported around 10% of opposite adaptation directions. This indicates that once an adaptation strategy is selected, it tends to be maintained across different tasks, although the magnitude of these adaptations may vary.

### Task-dependent neuromuscular adaptation to pain

The study found that the magnitude pain adaptation depends on the task being performed, confirming our hypothesis. The magnitude of muscle activation strategies in response to pain was smaller in tasks where the LEM had a mechanical advantage. Specifically, muscle activity amplitude and redistribution along the mediolateral axis were approximately 183 and 86% smaller, respectively, compared to a position of mechanical disadvantage. In the 45° trunk flexion position, the mechanical advantage of the muscle is hypothesized to facilitate the recruitment of LEM muscle fibers (Hudson et al., 2019) resulting in greater LEM muscle activation contribution compared to the 90° trunk flexion position (Abboud et al., 2023; Ducas et al., 2024). This increase in lumbar contribution would enable a more efficient load redistribution across muscle fibers to adapt to pain. This effective load redistribution would reduce the need for large-scale adaptations to pain. Consequently, in this position, our results suggest that subtle changes in LEM muscle activation patterns may be efficient in adapting to experimental pain. In contrast, in the 90° trunk flexion position, the mechanical disadvantage is hypothesized to inhibit the recruitment of LEM muscle fibers (Hudson et al., 2019), resulting in a decreased LEM muscle activation contribution (Ducas et al., 2024), which could limit their ability to adapt to the painful stimuli. Consequently, it could be hypothesized that compensatory recruitment of alternative lumbar or back extensor muscles may be necessary, resulting in a greater change in the muscle activation strategies measured in the lumbar region. Future research should build on these results by exploring more dynamic tasks.

### Magnitude of pain adaptations on motor performance

Greater magnitude of pain adaptations appears to have a direct negative relation on motor performance, as indicated by the inverse correlation between muscle redistribution magnitude along the mediolateral axis and force steadiness. This relation might be explained by the Uncontrolled Manifold hypothesis which suggests that the nervous system organizes and controls movement by separating task-relevant variability from task-irrelevant variability (Scholz and Schöner, 1999). Thus, the variability introduced by the nervous system to adapt to the painful stimulus might, depending on its magnitude, occur in dimensions that are task-relevant and critical to achieve the goal. This suggests that these compensatory pain adjustments may come at the cost of fine-tuned motor control. However, it is important to interpret this observation cautiously, particularly because the significance is limited to the left side and it is near the threshold for statistical significance. The observed asymmetry in this correlation may stem from the relatively lower force levels required, which might not significantly challenge the trunk musculature as a whole, as steadiness is predominantly influenced by the level of force exerted (Tracy et al., 2007; Schmidt et al., 1979). Consequently, individuals could effectively adapt to both the pain stimuli and maintain task-goal performance without encountering substantial difficulties. In contrast, tasks requiring greater effort could potentially trigger more pronounced motor consequences due to increased demands on the trunk musculature. Future studies should continue to explore the impact of neuromuscular adaptations magnitude on task-goal performance to better understand the nuanced relationship of the neuromuscular system adaptability and its consequence on motor performance. Moreover, the greater magnitude of neuromuscular adaptation to pain could contribute to sustained pain as pain adaptations have been observed to persist even after pain resolution (Summers et al., 2019; Tucker et al., 2012; Gallina et al., 2018) and that these compensatory adaptations are believed to contribute to the onset of chronic pain (MacDonald et al., 2009; Moseley and Hodges, 2006). Thus, future studies should aim to investigate whether a greater magnitude of pain adaptations to acute pain acts as a risk factor for the development of chronic pain.

### Clinical impact and future perspective

The high inter-individual variability observed in adaptations could explain the heterogeneity observed in studies on clinical low back pain (Van Dieën et al., 2019), which limits our understanding of this condition and makes treatment more challenging. This variability is likely more pronounced in individuals with chronic low back pain, where additional factors like changes in muscle structure (Matheve et al., 2023) and psychological factors (Watson et al., 1997; Geisser et al., 2004) can further influence pain adaptations. These findings highlight the importance of personalized treatment plans, where interventions are customized to each patient’s unique neuromuscular response to pain.

The task-dependent pain adaptation found in this study suggests that clinical interventions should account for the specific tasks patients are performing when addressing pain-related neuromuscular adaptations. By tailoring rehabilitation exercises to enhance mechanical advantages in particular tasks, clinicians may reduce compensatory strategies that impair motor performance, potentially preventing the persistence and the development of chronic pain. However, further research is needed to validate this approach. Future research should also investigate the impact of neuromuscular adaptation magnitude on task performance in tasks requiring higher effort levels (Arvanitidis et al., 2022).

### Limitations

First, the adaptations to pain were observable in tasks that required minimal effort, which is relevant for everyday activities. However, further investigation is required to determine if comparable variable adaptations occurred in tasks that require greater physical effort, potentially exerting a more substantial impact on performance.

In addition, our study did not assess the gluteus maximus and hamstring muscles due to methodological constraints imposed by the apparatus setup. Future research should include the measurement of these muscles, as lumbar pain adaptations may extend to the other back extensor muscles, providing a more comprehensive understanding of the observed adaptations of the lumbar region.

Moreover, our study’s findings may be limited by the specifics of our experimental pain model or the moderate level of induced pain (rated 3/10). Nevertheless, comparable findings were documented at higher pain levels, reaching a peak of 6/10, in a study by Hodges et al. (2013) using hypertonic saline injection into the muscle.

Finally, a significant limitation of current experimental pain models is their inability to adjust nociceptive input based on the neuromuscular adaptations chosen by the individual. Since the nociceptive input remains constant regardless of the adaptation, this could result in variable adaptations, as seen in this and other studies (Gallina et al., 2018; Hodges et al., 2013), because none may effectively alleviate pain. Future research should focus on developing innovative experimental pain models that allow real-time modulation of nociceptive input based on neuromuscular adaptations. This would enable a better understanding of how pain adaptations influence pain perception, rather than the opposite.

## Conclusion

In conclusion, this study highlighted the intricate and task-dependent nature of neuromuscular adaptations to pain within the lumbar muscles. The results illustrated inter-individual variability in neuromuscular adaptations in response to lumbar pain, highlighting distinct adaptation patterns. While pain adaptations tended to occur consistently between tasks, greater magnitude of adaptations were observed during mechanical disadvantage, which could have a direct impact on motor performance. These findings indicate a potential trade-off between pain adaptations and performance, suggesting that clinical interventions should consider the specific task patients are performing when addressing pain-related neuromuscular adaptations. Future research should explore the task-dependent nature of these adaptations in clinical populations to better understand their implications for motor performance and pain management.

## Data Availability

The datasets analyzed for this study can be found in the Borealis repository [https://doi.org/10.5683/SP3/QZJYO8].
